# Genomic features, phylogenetic relationships, and comparative genomics of *Elizabethkingia anophelis* strain EM361-97 isolated in Taiwan

**DOI:** 10.1038/s41598-017-14841-8

**Published:** 2017-10-30

**Authors:** Jiun-Nong Lin, Chung-Hsu Lai, Chih-Hui Yang, Yi-Han Huang, Hsi-Hsun Lin

**Affiliations:** 1 Department of Critical Care Medicine, E-Da Hospital, I-Shou University, Kaohsiung, Taiwan; 2 Division of Infectious Diseases, Department of Internal Medicine, E-Da Hospital, I-Shou University, Kaohsiung, Taiwan; 3 School of Medicine, College of Medicine, I-Shou University, Kaohsiung, Taiwan; 4 Department of Biological Science and Technology, Meiho University, Pingtung, Taiwan

## Abstract

*Elizabethkingia anophelis* has become an emerging infection in humans. Recent research has shown that previous reports of *E. meningoseptica* infections might in fact be caused by *E. anophelis*. We aimed to investigate the genomic features, phylogenetic relationships, and comparative genomics of this emerging pathogen. *Elizabethkingia anophelis* strain EM361-97 was isolated from the blood of a cancer patient in Taiwan. The total length of the draft genome was 4,084,052 bp. The whole-genome analysis identified the presence of a number of antibiotic resistance genes, which corresponded with the antibiotic susceptibility phenotype of this strain. Based on the average nucleotide identity, the phylogenetic analysis revealed that *E. anophelis* EM361-97 was a sister group to *E. anophelis* FMS-007, which was isolated from a patient with T-cell non-Hodgkin’s lymphoma in China. Knowledge of the genomic characteristics and comparative genomics of *E. anophelis* will provide researchers and clinicians with important information to understand this emerging microorganism.

## Introduction

*Elizabethkingia* is a genus of aerobic, nonfermenting, nonmotile, catalase-positive, oxidase-positive, indole-positive, and gram-negative bacilli that are usually distributed in soil and water environments^1–3^. Genus *Elizabethkingia* has been reported to cause human infection since Elizabeth O. King’s original work in 1959^4^. However, this genus had rarely been responsible for infections in humans before. These microorganisms have been recently reported to cause life-threatening infections in immunocompromised patients, such as pneumonia, bacteraemia, meningitis, and neutropenic fever^1–7^.

Among genus *Elizabethkingia*, *E. meningoseptica*, previously known as *Chryseobacterium meningosepticum*, is the most well-known species that causes opportunistic infection in humans^2,3^. In contrast, little is known about *E. anophelis*. *Elizabethkingia anophelis* was first isolated from the midgut of a mosquito, *Anopheles gambiae*, in 2011^8^ and has caused several outbreaks of infections in Africa^7,9^, Singapore^10^, Hong Kong^11^, and the USA^5,6,12^. The Centers for Disease Control and Prevention of the USA reported two outbreaks of infections caused by *E. anophelis* in the Midwest. A total of 63 patients in Wisconsin were confirmed to have *E. anophelis* infection between November 1, 2015 and April 12, 2017, and this outbreak caused 19 deaths^13^. Another cluster of 10 patients with *E. anophelis* infection was reported in Illinois, and six of the patients died of this infection^5^. Pulsed-field gel electrophoresis and whole-genome sequencing revealed that the strains of *E. anophelis* in these two outbreaks were genetically different^6^. However, recent research has shown that *E. anophelis* was frequently misidentified as *E. meningoseptica*, and previous reports of *E. meningoseptica* infections might in fact be caused by *E. anophelis* ^9–12^.

We previously published the draft whole-genome sequence of *E. anophelis* strain EM361-97 isolated in Taiwan (GenBank accession number, LWDS00000000.1)^14^. The whole-genome sequence could provide insights into the characteristics of the putative virulence factors, pathogenesis, and drug resistance of microorganisms. Comparison of genomes among different strains can be used in the analyses of phylogenetic relationships and epidemiological features. However, there has been little research investigating the genomic characteristics, global epidemiology, and genomic diversity of *E. anophelis*. In this study, we analysed the genomic features of *E. anophelis* strain EM361-97. We also compared the genomics and investigated the phylogenetic relationships with other strains of *E. anophelis* from other world regions.

## Materials and Methods

### Ethics and experimental biosafety statements

This study was approved by the Institutional Review Board of E-Da Hospital (EMRP-105-134). The need for patient’s informed consent was waived by the Institutional Review Board of E-Da Hospital as the retrospective analysis of anonymously clinical data posed no more than minimal risk of harm to subjects and involved no procedures for which written consent was normally required outside of the research context. The experiments in this study were approved by the Institutional Biosafety Committee of E-Da Hospital. All experiments were performed in accordance with relevant guidelines and regulations.

### Isolate of *E. anophelis*

*Elizabethkingia anophelis* strain EM361-97 was isolated from the blood of a 46-year-old male patient with advanced nasopharyngeal carcinoma and lung cancer. During admission, the patient suffered from pneumonia, respiratory failure, and profound shock. He initially received empirical antibiotics with levofloxacin. Unfortunately, the patient died several days after this infection. One blood culture from the patient yielded a gram-negative bacillus that was initially identified as *E. meningoseptica* using API/ID32 GN (bioMérieux S.A., Marcy l’Etoile, France) by the clinical microbiology laboratory. This isolate was named strain EM361-97 and was stored at −80 °C as a glycerol stock for further experiments. We re-identified this isolate as *E. anophelis* using 16S ribosomal RNA (rRNA) gene sequencing as previously published^15^. The minimum inhibitory concentration (MIC) of this isolate was examined using the broth microdilution method. The susceptibilities were determined according to the interpretive standards for “other non-*Enterobacteriaceae*” as suggested by the Clinical and Laboratory Standards Institute (CLSI) guidelines^16^.

### Whole-genome sequencing and genome annotation of *E. anophelis* EM361-97

The deoxyribonucleic acid (DNA) of this isolate was prepared using a Wizard Genomic DNA Purification Kit according to the manufacturer’s instructions (Promega, WI, USA). The genome was sequenced using an Illumina HiSeq. 2000 Sequencing Platform (Illumina, CA, USA). The short reads were assembled and optimized according to paired-end and overlap relationship via mapping reads to contig using SOAP de novo v. 2.04^17^. The assembled genome was then submitted to the NCBI Prokaryotic Genome Annotation Pipeline^18^ and the Rapid Annotations based on Subsystem Technology (RAST) Prokaryotic Genome Annotation Server (http://rast.nmpdr.org/) for gene function annotations^19,20^. The graphical map of the circular genome was generated using the CGView Server (http://stothard.afns.ualberta.ca/cgview_server/)^21^. The virulence factors of strain EM361-97 were analysed using the Virulence Factor Database (VFDB, http://www.mgc.ac.cn/VFs/)^22,23^. Antibiotic resistance genes were searched using the Antibiotic Resistance Genes Database BLAST Server (https://ardb.cbcb.umd.edu/)^24^, RAST Server^19,20^, and UniProtKB/Swiss-Prot database via OrthoVenn (http://probes.pw.usda.gov/OrthoVenn/)^25^.

### Comparative genomic analysis

For comparison, the genome sequences of 34 available, nonduplicated, different genome sequences of *E. anophelis* in GenBank were downloaded from the National Center for Biotechnology Information (NCBI) genome sequence repository (https://www.ncbi.nlm.nih.gov/genome/). The genome-wide comparison and annotation of clusters of orthologous groups (COGs) were generated using the web server OrthoVenn^25^. The average nucleotide identity (ANI) values between two genome sequences were calculated using the original ANI function of OrthoANI^26^. The heat maps were generated using CIMminer (https://discover.nci.nih.gov/cimminer/). The *in silico* DNA-DNA hybridization (DDH)-analogous values between different strains were calculated using the Genome-to-Genome Distance Calculator (GGDC) 2 (http://ggdc.dsmz.de/distcalc2.php)^27^. A 70% similarity of *in silico* DDH value represents the cut-off value for species boundaries. The phylogenetic tree was constructed using CIMminer (https://discover.nci.nih.gov/cimminer/) based on ANI values.

### Data Availability

The names of organisms, strains, biosample numbers, bioproject numbers, assembly numbers, isolated origins, and release dates of bacteria used in this study are shown in Supplementary Table S1.

## Results and Discussion

### General genome description of *E. anophelis* EM361-97

**Figure 1:**
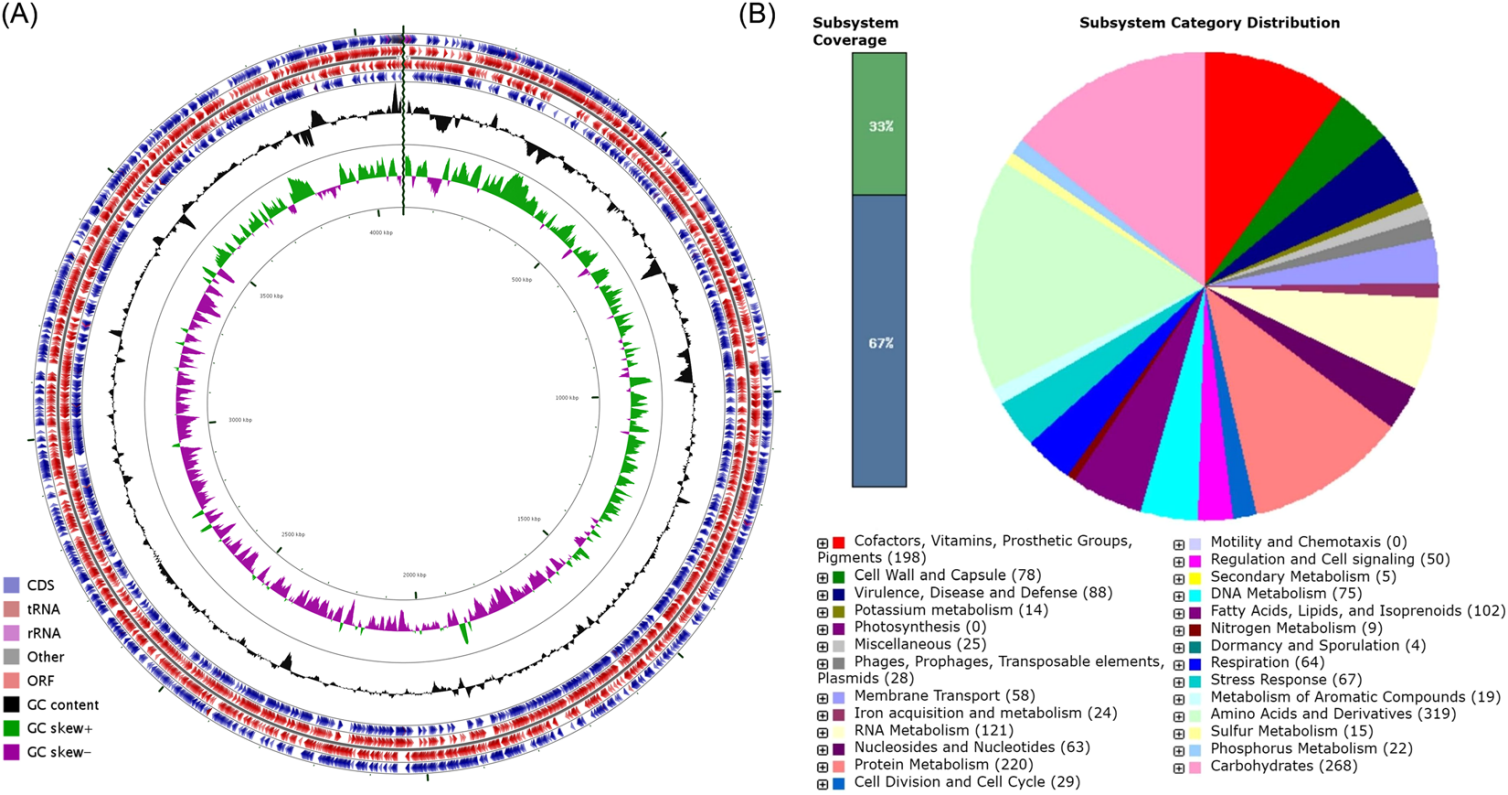
Circular representation and subsystem category distribution of the genome of *E. anophelis* EM361-97. (**A**) Circles are numbered from 1 (the outermost circle) to 7 (the innermost circle). The outer four circles show the coding sequence (CDS), transfer ribonucleic acid (tRNA), ribosomal ribonucleic acid (rRNA), and open reading frame (ORF). The fifth circle represents the GC content (black). The sixth circle demonstrates the GC skew curve (positive GC skew, green; negative GC skew, violet). The genome position scaled in kb from base 1 is shown on the inner circle. (**B**) The genome of *E. anophelis* EM361-97 annotated using the Rapid Annotation System Technology (RAST) Server was classified into 356 subsystems and 27 categories. The green part in the bar chart at the leftmost position corresponds to the percentage of proteins included. The pie chart and the count of subsystem features in the right panel demonstrate the percentage distribution and category of the subsystems in *E. anophelis* EM361-97.

The statistics of assembly and annotation are shown in Table 1. The total length of the draft genome was 4,084,052 bp, with a mean GC content of 35.7%. This genome contained 3,774 genes that made up 87.9% of genome. The genomic features of *E. anophelis* EM361-97 are shown in Fig. 1. The number of tandem repeat sequence was 108. The assembly contained 18 scaffolds, 27 contigs, 3,743 coding sequences (CDSs), 53 minisatellite DNAs, 26 microsatellite DNAs, 51 transfer RNAs (tRNAs), and 15 rRNAs (Fig. 1A).

**Table 1:** Assembly and annotation statistics.

Total sequence length (bp)	4,084,052
Total assembly gap length (bp)	6,353
Gaps between scaffolds	0
Number of scaffolds	18
Scaffold N50 (bp)	4,056,868
Scaffold L50	1
Number of contigs	27
Contig N50 (bp)	1,882,703
Contig L50	2
Read length (bp)	100
Coverage depth	95.95
GC content (%)	35.7

The genomic features of microorganism could be investigated according to the subsystem, a cluster of genes that function with a specific biological process or structural complex^19,20^. The genome of *E. anophelis* strain EM361-97 analysed by the RAST Server revealed 356 subsystems that could be classified into 27 categories (Fig. 1B). Among these, the “amino acid and derivatives” subsystem accounted for the largest number of 319 CDSs, followed by carbohydrate metabolism (268 CDSs), protein metabolism (220 CDSs), and RNA metabolism (121 CDSs). Regarding the 88 CDSs in the “virulence, disease, and defense” subsystem, 12 were related to invasion and intracellular resistance, and 76 were associated with resistance to antibiotics and toxic compounds. The high number of antibiotic resistance-associated CDSs suggests that *E. anophelis* EM361-97 might be resistant to multiple antibiotics.

### Orthologous genes

**Figure 2:**
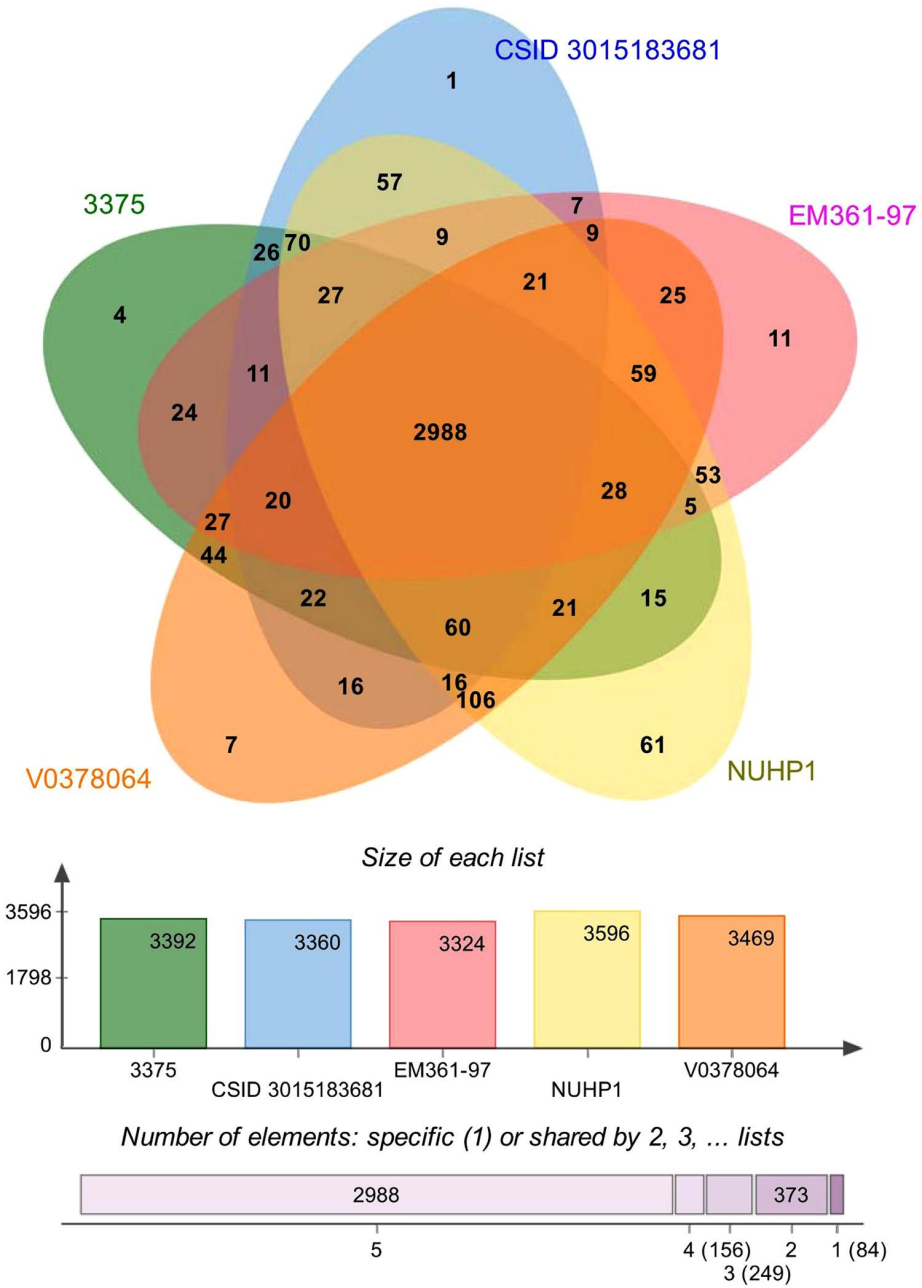
Proteome comparison among *E. anophelis* strains EM361-97 (origin, Taiwan), CSID_3015183681 (origin, USA), 3375 (origin, USA), V0378064 (E18064) (origin, Africa), and NUHP1 (origin, Singapore). The Venn diagram and bar chart represent the numbers of unique and shared orthologous genes of each strain.

Orthologous genes are clusters of genes in different species that have evolved by vertical descent from a single ancestral gene. A genome-wide comparison of orthologous clusters in different isolates provides insight into the gene structure, gene function, and molecular evolution of genomes^25^. The COGs analysis of strain EM361-97 was compared with the other four genomes isolated from the USA (strains CSID_3015183681 and 3375), Africa (strain V0378064 [E18064]), and Singapore (strain NUHP1) (Fig. 2). The analysis shows that *E. anophelis* EM361-97 contained 3,611 proteins, 3,324 COGs, and 234 singletons. Among the 3,324 COGs in strain EM361-97, 2,988 COGs were shared by all five strains, and 11 COGs were only present in the strain EM361-97 genome. The unique COGs existing in EM361-97 involved genes functioning with transferase activity, cofactor binding, oxidoreductase activity, nucleotide binding, fatty acid elongation, and 3-oxoacyl-[acyl-carrier-protein] reductase (NADPH) activity. The representative meanings of these singular genes in *E. anophelis* EM361-97 are not clear. Further investigations to understand the features of these unique genes in *E. anophelis* EM361-97 are warranted.

### Genomic comparison among *Elizabethkingia* species

**Figure 3:**
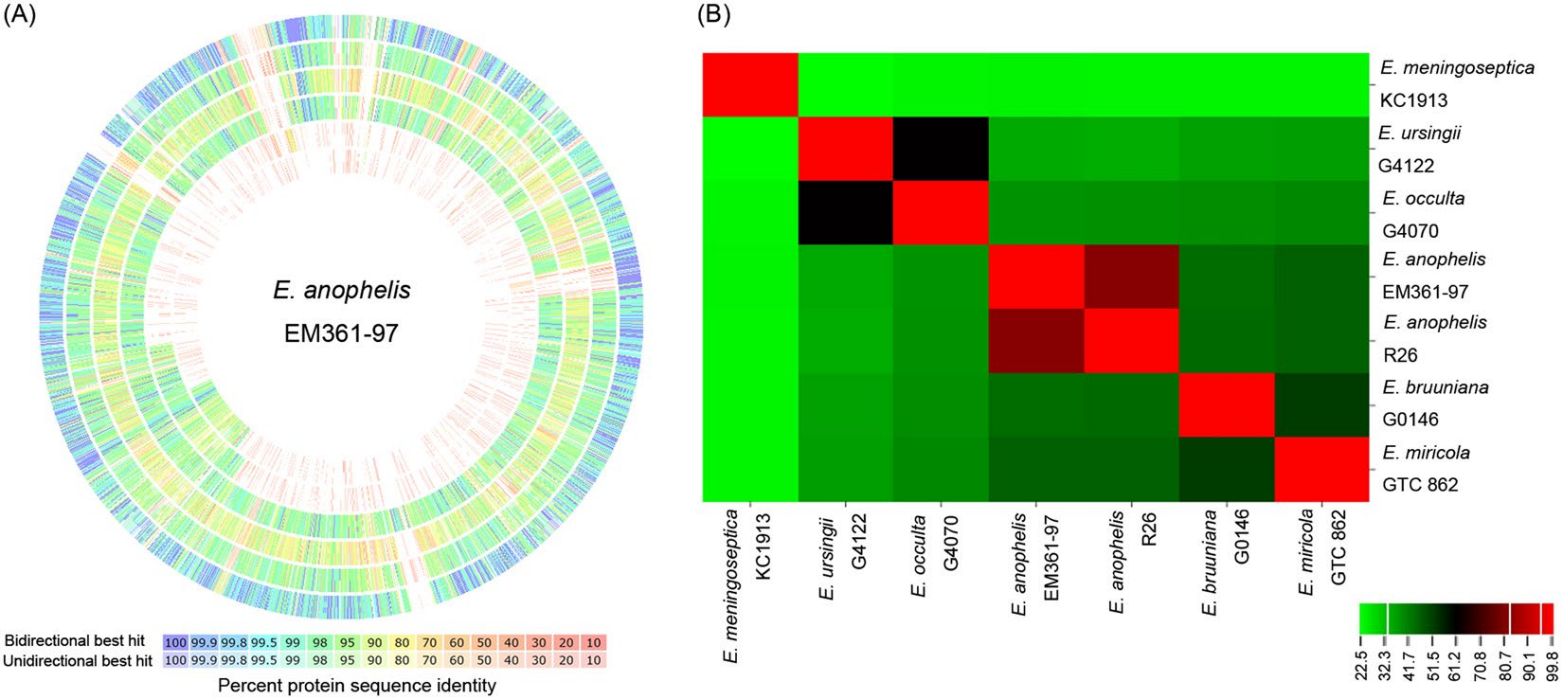
Genomic comparison among *Elizabethkingia* species. (**A**) The genome of *E. anophelis* EM361-97 (center) compared to *E. anophelis* R26^T^ (the outermost circle; ring 1), *E. bruuniana* G0146^T^ (ring 2), *E. meningoseptica* KC1913^T^ (ring 3), *E. miricola* GTC 862 ^T^ (ring 4), *E. occulta* G4070^T^ (ring 5), and *E. ursingii* G4122^T^ (the innermost circle; ring 6). The genome of *E. anophelis* EM361-97 was highly similar to the type strain of *E. anophelis* R26^T^. (**B**) The *in silico* DNA-DNA-hybridization (DDH) values between different strains calculated using the Genome-to-Genome Distance Calculator. The DDH value between *E. anophelis* EM361-97 and *E. anophelis* R26^T^ was 82%. *Elizabethkingia meningoseptica* KC1913^T^ demonstrated a relatively large phylogenetic distance from other strains of *Elizabethkingia*.

The genomic comparison among *E. anophelis* EM361-97, *E. anophelis* R26^T^, *E. meningoseptica* KC1913^T^, *E. miricola* GTC 862 ^T^, *E. bruuniana* G0146^T^, *E. ursingii* G4122^T^, and *E. occulta* G4070^T^ implemented using the RAST/SEED Server is shown in Fig. 3A. The genome of *E. anophelis* EM361-97 was apparently closer to that of *E. anophelis* R26^T^ than the other *Elizabethkingia* species. The evolutionary relatedness among these strains was measured by *in silico* DDH-analogous values (Fig. 3B). The DDH value between *E. anophelis* EM361-97 and *E. anophelis* R26^T^ was 82%. In contrast, the DDH value between *E. anophelis* EM361-97 and *E. meningoseptica* KC1913^T^ was only 24.2%.

Genus *Elizabethkingia* previously comprised four species, namely *E. meningoseptica*, *E. miricola*, *E. anophelis*, and *E. endophytica* ^28^. However, the strain of *E. endophytica* was re-identified as an additional strain of *E. anophelis* based on *in silico* DDH of whole-genome sequencing (77% DDH value with regard to *E. anophelis* strain R26^T^)^29^. Recently, Nicholson *et al*.^30^ proposed three novel *Elizabethkingia* species, *Elizabethkingia bruuniana* sp. nov., *Elizabethkingia ursingii* sp. nov., and *Elizabethkingia occulta* sp. nov. Our study showed that strain EM361-97 belonged to *E. anophelis*, with a DDH value of 82% between *E. anophelis* EM361-97 and the type strain of *E. anophelis* R26^T^. In addition, *Elizabethkingia meningoseptica* KC1913^T^ demonstrated a relatively large phylogenetic distance from other strains of *Elizabethkingia*. These findings are consistent with the previous report of the taxonomic classification in genus *Elizabethkingia* ^30^.

### Whole-genome phylogenetic analysis of *E. anophelis*

**Figure 4:**
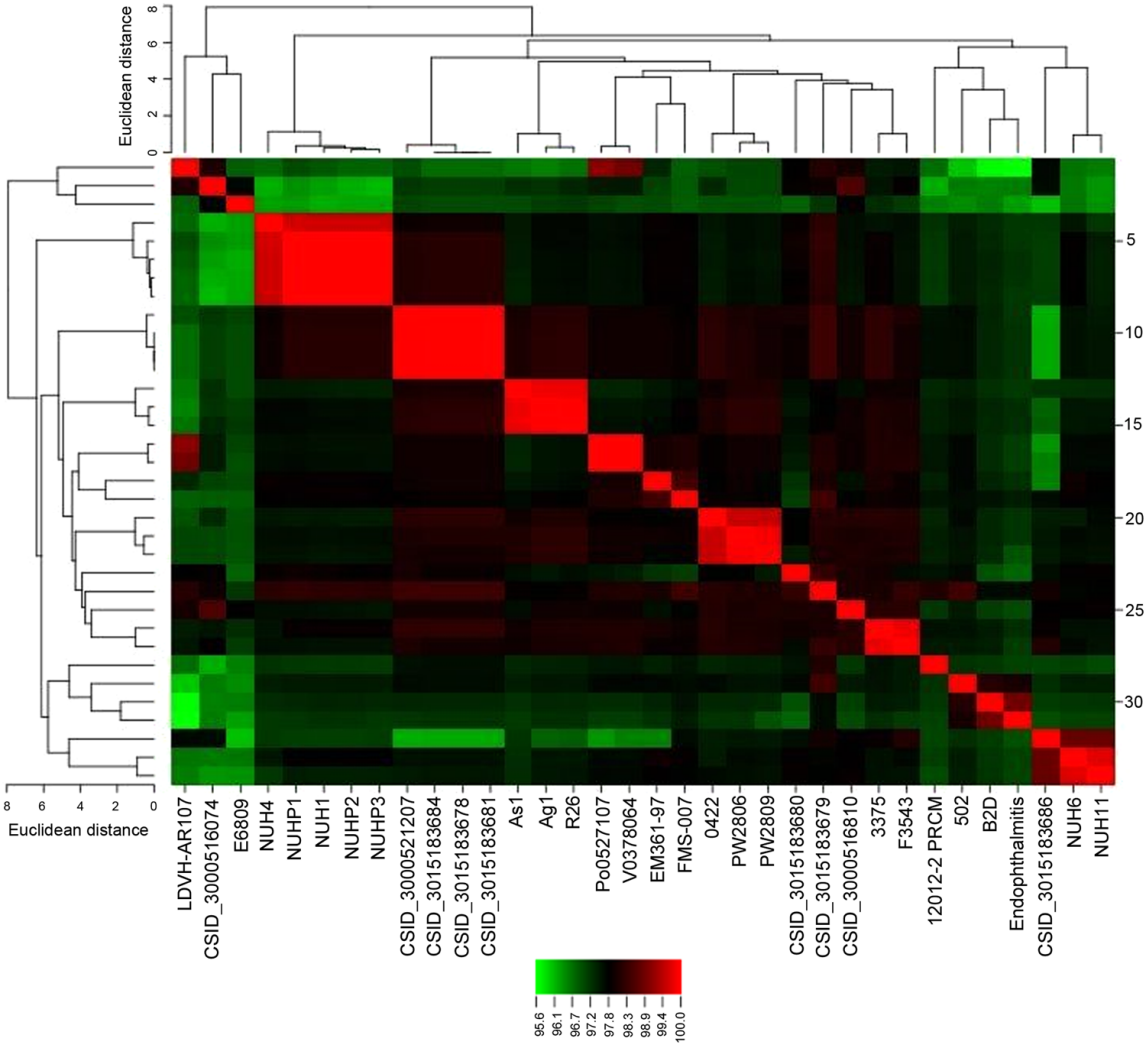
The phylogenetic tree of the 34 available strains of *E. anophelis* in GenBank based on average nucleotide identity (ANI) values. The phylogenetic analysis revealed that *E. anophelis* EM361-97 was a sister group to *E. anophelis* FMS-007, which was a clade sister of strains Po0527107 (E27017) and V0378064 (E18064) isolated in the Central African Republic.

The phylogeny of the 34 available strains of *E. anophelis* based on ANI is shown in Fig. 4. The phylogenetic analysis revealed that *E. anophelis* EM361-97 was a sister group to *E. anophelis* FMS-007, which was isolated from a patient with T-cell non-Hodgkin’s lymphoma in China. The sister group of *E. anophelis* strains EM361-97 and FMS-007 was a clade sister of strains Po0527107 (E27017) and V0378064 (E18064) isolated from two neonates with meningitis in the Central African Republic^7^. The seven strains isolated from Singapore were divided into two clusters (NUHP1, NUHP2, NUHP3, NUH1, NUH4; and NUH6, NUH11). The 13 strains isolated from the USA clustered in four groups, and the four strains that caused the outbreak of *E. anophelis* infection in Wisconsin (strains CSID_3015183678, CSID_3015183681, CSID_3015183684, CSID_3000521207) were in the same clade.

### Virulence factors

*Elizabethkingia anophelis* infections in humans have shown a mortality rate of 24% to 60%^5,6,11^, and this high mortality rate may be in part correlated with the virulence of this pathogen and also the preexisting conditions of the patients (*e.g*., old age, neonates, and immunosuppression). In this study, homologs of 25 virulence factors were identified in *E. anophelis* EM361-97 using VFDB^22,23^ (Supplementary Table S2). These virulence factors included products of the capsule, lipopolysaccharide, endopeptidase, lipid biosynthesis and metabolism, magnesium transport protein, macrophage infectivity, heat shock protein, catalase, peroxidase, superoxide dismutase, two-component regulatory system, and others.

According to the VFDB classification scheme, virulence factors are divided into offensive, defensive, nonspecific, and virulence-associated regulatory genes^22^. In our study, 13 of 25 pathogen-associated virulence factors homologs were identified to play offensive functional roles, eight were associated with defensive functions, three were nonspecific virulence factors, and one was related to regulation of virulence-associated genes. In strains Po0527107 (E27017) and V0378064 (E18064), Breurec *et al*.^7^ identified several offensive virulence factors that were found in strain EM361-97, including *clpC*, *kdtB*, *pilR*, *sodB*, *galE*, *bplC*, *katA*, *clpP*, *fleQ*, and *htpB*. These virulence factors were also detected in the Wisconsin strains^12^.

Pathogenic genomes were identified to have more offensive virulence factors, such as toxin and type III/IV secretion systems, than non-pathogenic genomes. In contrast, defensive, nonspecific, and regulatory virulence factors, such as iron uptake, motility, and antiphagocytosis, were found more frequently in non-pathogenic genomes than in pathogenic genomes^31^. Ho Sui *et al*.^32^ carried out a large-scale study to analyse the virulence factors of multiple bacteria and found over-presentation of offensive virulence factors, such as type III/IV secretion systems or toxins, within genomic islands of invasive pathogens. The manifestation of many offensive virulence factors in *E. anophelis* suggests this microorganism may severely damage the host. However, this hypothesis lacks validity. More experiments are warranted to test the hypothesis of offensive virulence factors in *E. anophelis*.

### Antimicrobial resistance and associated genes of *E. anophelis* EM361-97

The MIC and susceptibility of *E. anophelis* EM361-97 are shown in Table 2. This isolate was only susceptible to piperacillin-tazobactam and minocycline. The MIC of tigecycline was 2 mg/L. However, there are no interpretive criteria of the susceptibility for *E. anophelis* to tigecycline in the CLSI^16^ and European Committee on Antimicrobial Susceptibility Testing^33^.

**Table 2:** The minimum inhibitory concentration, susceptibility, and genes associated with antibiotic resistance in *E. anophelis* EM361-97.

Antibiotic Group^†^	Antibiotics	MIC	Interpretation^*^	Resistant Gene/Protein/Mechanism^†^
Penicillins	Piperacillin	32	I	β-lactamase (BRO-1, 2)Class A β-lactamaseGOB-1 β-lactamaseSubclass B3 metallo-β-lactamaseSubclass B1 metallo-β-lactamase
β-lactam/β-lactamase inhibitor combinations	Piperacillin-tazobactam	16/4	S	
	Ticarcillin-clavulanic acid	>64/2	R	
Cephems	Ceftazidime	>16	R	
	Cefepime	32	R	
	Ceftriaxone	>32	R	
Monobactams	Aztreonam	>16	R	
Carbapenems	Imipenem	>8	R	Carbapenem antibiotics biosynthesis protein CarD
	Meropenem	>8	R	
Aminoglycosides	Gentamicin	>8	R	Resistance-nodulation-cell division transporter system Multidrug resistance efflux pumpAminoglycoside N-acetyltransferaseAPH(3’) family aminoglycoside O-phosphotransferaseelongation factor Tu
	Tobramycin	>8	R	
	Amikacin	>32	R	
Tetracyclines	Tetracycline	>8	R	Tetracycline resistance protein TetXMajor facilitator superfamily transporterTetracycline efflux pumpNADP-requiring oxidoreductase
	Minocycline	<1	S	
	Tigecycline	2	—	
Fluoroquinolones	Ciprofloxacin	>2	R	DNA gyrase subunit A and subunit BTopoisomerase IV
	Levofloxacin	>8	R	
Folate pathway inhibitors	Trimethoprim-sulfamethoxazole	>4/76	R	Group A drug-insensitive dihydrofolate reductaseSulfonamide-resistant dihydropteroate synthase Sul1
Macrolides	—	—	—	Macrolide export protein MacA, MacBErythromycin resistance ATP-binding protein MsrA
Vancomycin	—	—	—	Vancomycin B-type resistance protein VanW
Clindamycin	—	—	—	Lincomycin resistance protein
Chloramphenicol	—	—	—	Resistance-nodulation-cell division transporter system Multidrug resistance efflux pumpGroup B chloramphenicol acetyltransferase (xenobiotic acetyltransferase)

MIC, minimum inhibitory concentration.

^†^Associated with multidrug resistance: membrane component of tripartite multidrug resistance system, multidrug resistance efflux pumps (CmeB, TolC, MATE family efflux pump, OML, AcrA, AcrB), outer membrane efflux protein BepC, multidrug resistance proteins (MdtA, MdtB, MdtC, MdtD, MdtE, MdtK, MdtL), multidrug resistance protein EmrK, multidrug export protein EmrA, multidrug resistance outer membrane protein MdtQ, ABC transporter, MFS transporter, transcription-repair coupling factor, acriflavin resistance protein, and isoleucine-tRNA ligase.

^*^Susceptibility was determined according to the interpretive standards for other non*-Enterobacteriaceae* of CLSI.

Little information is known about the antimicrobial susceptibility of *E. anophelis*. Han *et al*.^34^ reported the susceptibilities of 51 *E. anophelis* isolates from South Korea. The susceptibility rates to piperacillin-tazobactam, piperacillin, levofloxacin, ciprofloxacin, gentamicin, and trimethoprim-sulfamethoxazole were 92%, 82%, 29%, 22%, 22%, and 22%, respectively. All the isolates were resistant to ceftazidime and imipenem. However, the MICs of minocycline and tigecycline were not examined in that study^34^. Perrin *et al*.^12^ used the disk diffusion method to examine antimicrobial susceptibilities of 29 *E. anophelis* isolates in the Wisconsin outbreak. Most of these isolates were resistant to ceftazidime, imipenem, amikacin, tobramycin, gentamicin, but susceptible to cefepime, piperacillin, piperacillin-tazobactam, ciprofloxacin, and levofloxacin. Minocycline was also not tested in the study of Perrin *et al*.^12^. The antibiogram of isolates in the Wisconsin outbreak was different from that of isolates in Singapore by macrolides and isepamycin^10,35^.

Gene functions annotated using the RAST/SEED Server recognised 76 genes of *E. anophelis* EM361-97 that were related to antibiotic resistance, including 12 for β-lactamase resistance, one for vancomycin resistance (*vanW*), four for fluoroquinolone resistance (*parC*, *parE*, *gyrA*, *gyrB*), nine for the membrane component of the tripartite multidrug resistance system, and 16 for multidrug resistance efflux pumps (six CmeB, one TolC, two MATE family efflux pumps, five OML, and two AcrB) (Table 2). The protein function annotations based on UniProtKB/Swiss-Prot demonstrated a number of proteins that played the role of antibiotic resistance, including multidrug resistance proteins (MdtA, MdtB, MdtC, MdtD, MdtE, MdtK, MdtL), probable multidrug resistance protein EmrK, multidrug export protein EmrA, macrolide export protein MacA, macrolide export ATP-binding/permease protein MacB, multidrug resistance outer membrane protein MdtQ, outer membrane efflux protein BepC, carbapenem antibiotics biosynthesis protein CarD, β-lactamase (BRO-1, 2), multidrug efflux pump subunit AcA, lincomycin resistance protein, DNA gyrase subunit A and subunit B, erythromycin resistance ATP-binding protein MsrA, and vancomycin B-type resistance protein VanW (Table 2). A replacement of serine by isoleucine at position 83 of DNA gyrase subunit A (Ser83Ile; AGC → ATC) was identified in *E. anophelis* strain EM361-97. Perrin *et al*.^12^ also found the same mutation of DNA gyrase subunit A in the Wisconsin outbreak strain CSID_3000521792. These findings are in agreement with the resistance of these two strains to fluoroquinolones.

## Conclusions

In this work, the genomic features of the *E. anophelis* strain EM361-97 were constructed and compared with the genomes of other *Elizabethkingia* strains. Functional studies of this pathogen are required to validate these findings.

## Electronic supplementary material

Supplementary Tables
