# Supplementary material for: Genomic features, phylogenetic relationships, and comparative genomics of Elizabethkingia anophelis strain EM361-97 isolated in Taiwan
Source: Sci Rep. 2017 Oct 30;7:14317. doi: 10.1038/s41598-017-14841-8 (PMC5662595; doi:10.1038/s41598-017-14841-8)
Supplement: Supplementary file 1 — Supplementary Tables [file 41598_2017_14841_MOESM1_ESM.pdf]

Genomic features, phylogenetic relationships, and comparative genomics of *Elizabethkingia anophelis* strain EM361-97 isolated in Taiwan

Jiun-Nong Lin[1,2,3], Chung-Hsu Lai[2], Chih-Hui Yang[4], Yi-Han Huang[1] & Hsi-Hsun Lin[2]

[1]Department of Critical Care Medicine, E-Da Hospital, I-Shou University, Kaohsiung, Taiwan. [2]Division of Infectious Diseases, Department of Internal Medicine, E-Da Hospital, I-Shou University, Kaohsiung, Taiwan. [3]School of Medicine, College of Medicine, I-Shou University, Kaohsiung, Taiwan. [4]Department of Biological Science and Technology, Meiho University, Pingtung, Taiwan.

Corresponding author: Jiun-Nong Lin, MD, PhD

Department of Critical Care Medicine, E-Da Hospital, I-Shou University, Kaohsiung, Taiwan

No. 1, Yida Road, Jiaosu Village, Yanchao District, Kaohsiung, Taiwan

E-mail: jinoli@kmu.edu.tw

Tel.: +886-7-6150011-251469

Fax: +886-7-615-0928

Key words: *Elizabethkingia anophelis*, genome, virulence factor, antibiotic resistance, epidemiology.

Table S1. The name of organism, strain, biosample number, bioproject number, assembly number, isolated origin, and release date of bacteria used in this study.

Organism/Name	Strain	BioSample	BioProject	Assembly	Origin	Release Date
Chryseobacterium indologenes	NBRC 14944	SAMD00041821	PRJDB276	GCA_000520835.1	Japan	2014/01/11
Elizabethkingia meningoseptica	ATCC 13253	SAMN02470766	PRJNA198814	GCA_000401415.1	USA	2013/05/24
Elizabethkingia miricola	BM10	SAMN03398457	PRJNA277897	GCA_000955665.1	Korea	2015/03/17
Elizabethkingia anophelis	Ag1	SAMN02470679	PRJNA77829	GCA_000240095.2	Africa	2011/12/23
Elizabethkingia anophelis	502	SAMN02471026	PRJNA176121	GCA_000447375.1	UK	2013/8/15
Elizabethkingia anophelis	NUH1	SAMN02470987	PRJNA205480	GCA_000495995.1	Singapore	2013/11/7
Elizabethkingia anophelis	NUH11	SAMN02470986	PRJNA205483	GCA_000496055.1	Singapore	2013/11/7
Elizabethkingia anophelis	NUH4	SAMN02470992	PRJNA205481	GCA_000496015.1	Singapore	2013/11/7
Elizabethkingia anophelis	NUH6	SAMN02470990	PRJNA205482	GCA_000496035.1	Singapore	2013/11/7
Elizabethkingia anophelis	NUHP2	SAMN02470989	PRJNA205478	GCA_000495955.1	Singapore	2013/11/7
Elizabethkingia anophelis	NUHP3	SAMN02470991	PRJNA205479	GCA_000495975.1	Singapore	2013/11/7
Elizabethkingia anophelis	Po0527107 (E27017)	SAMEA3139014	PRJEB5243	GCA_000689515.1	Africa	2014/4/10
Elizabethkingia anophelis	V0378064 (E18064)	SAMEA3139011	PRJEB5242	GCA_000689455.1	Africa	2014/4/10
Elizabethkingia anophelis	B2D	SAMN02797821	PRJNA248328	GCA_000735205.1	Malaysia	2014/7/28
Elizabethkingia anophelis	NUHP1	SAMN02470988	PRJNA205476	GCA_000495935.2	Singapore	2014/8/27
Elizabethkingia anophelis	Endophthalmitis	SAMN03107819	PRJNA263868	GCA_000773465.1	India	2014/11/7
Elizabethkingia anophelis	PW2806	SAMEA3158465	PRJEB5024	GCA_000982575.1	Hong Kong	2015/1/15
Elizabethkingia anophelis	PW2809	SAMEA3158491	PRJEB5025	GCA_001050935.1	Hong Kong	2015/2/2
Elizabethkingia anophelis	FMS-007	SAMN03704061	PRJNA210135	GCA_001011675.1	China	2015/5/21

Elizabethkingia anophelis	As1	SAMN03491119	PRJNA272632	GCA_001051975.1	Asia	2015/7/10
Elizabethkingia anophelis	12012-2 PRCM	SAMN04272205	PRJNA302576	GCA_001482795.1	China	2015/12/28
Elizabethkingia anophelis	CSID_3015183678	SAMN04567744	PRJNA301708	GCA_001596175.2	USA	2016/3/23
Elizabethkingia anophelis	CSID_3000521207	SAMN04567738	PRJNA315668	GCA_001618505.2	USA	2016/4/11
Elizabethkingia anophelis	CSID_3015183681	SAMN04567745	PRJNA315668	GCA_001618545.2	USA	2016/4/11
Elizabethkingia anophelis	CSID_3015183684	SAMN04590540	PRJNA315668	GCA_001618465.2	USA	2016/4/11
Elizabethkingia anophelis	EM361-97	SAMN04599207	PRJNA317034	GCA_001703835.1	Taiwan	2016/8/11
Elizabethkingia anophelis	LDVH-AR107	SAMEA4026800	PRJEB14302	GCA_900156945.1	Africa	2017/1/19
Elizabethkingia anophelis	422	SAMN04254539	PRJNA326741	GCA_002022025.1	USA	2017/3/6
Elizabethkingia anophelis	3375	SAMN05273152	PRJNA326741	GCA_002022065.1	USA	2017/3/6
Elizabethkingia anophelis	E6809	SAMN04482265	PRJNA301708	GCA_002023235.1	USA	2017/3/7
Elizabethkingia anophelis	CSID_3000516074	SAMN05255124	PRJNA301708	GCA_002023325.1	USA	2017/3/8
Elizabethkingia anophelis	CSID_3000516810	SAMN05256530	PRJNA301708	GCA_002023425.1	USA	2017/3/8
Elizabethkingia anophelis	CSID_3015183679	SAMN05275358	PRJNA301708	GCA_002023645.1	USA	2017/3/8
Elizabethkingia anophelis	CSID_3015183680	SAMN05275369	PRJNA301708	GCA_002023695.1	USA	2017/3/8
Elizabethkingia anophelis	CSID_3015183686	SAMN05277596	PRJNA301708	GCA_002023705.1	USA	2017/3/8
Elizabethkingia anophelis	R26	SAMN02470677	PRJNA301708	GCA_002023665.1	USA	2017/3/8
Elizabethkingia anophelis	F3543	SAMN04482309	PRJNA326741	GCA_002024825.1	Africa	2017/3/10

Table S2. Virulence factors of *E. anophelis* EM361-97 identified using Virulence Factor Database (VFDB).

No.	Subject ID	Identity	E-value	Virulence	Gene	Classification	Mechanism	Characteristics and function
1	VFG0079	50.2	0	ClpC	*clpC*	Offensive virulence factors	Stress protein	Endopeptidase Clp ATP-binding chain C, belonging to the Hsp100/Clp family. An ATPase promotes early escape form the phagosome of macrophages. ClpC is also required for adhesion and invasion.
2	VFG0320	44.3	1.00E-35	LPS	*kdtB*	Offensive virulence factors	Toxin Adherence	Lipopolysaccharide core biosynthesis protein. Mediate a lectin-like interaction with laminin. The binding may disrupt epithelial cell-basement membrane interactions contributing to the disruption of gastric mucosal integrity and the development of gastric leakiness associated with the bacterium.
3	VFG0869	44.81	2.00E-39	Dispersin	*aatC*	Offensive virulence factors	Adherence	AatC ATB binding protein of ABC transporter. Its presence in the vast majority of EAEC strains make it a potential candidate for vaccine development; exported by a putative ABC transporter complex encoded by a gene cluster designated aatPABCD. Typical signal sequence, secreted to the extracellular milieu, where it remains non-covalently attached to the surface of the bacterium. Promotes dispersal of EAEC on the intestinal

								mucosa to establish new foci of infection and facilitate efficient colonization via bacterial dispersal.
4	VFG1369	41.33	4.00E-40	Capsule	*cps4E*	Defensive virulence factors	Anti-phagocytosis	Capsular polysaccharide biosynthesis protein Cps4E. Ninety different capsule types have been identified. Each has a structurally distinct capsule, composed of repeating oligosaccharide units joined by glycosidic linkages. Resistant to complement deposition and masks cell wall-associated complement from being recognized by the complement receptors on phagocytes.
5	VFG1417	41.8	8.00E-49	PanC/PanD	*panC/panD*	Defensive virulence factors	Cellular metabolism	Lipid biosynthesis and metabolism play a pivotal role in the intracellular replication and persistence of *M. tuberculosis*. Pantothenic acid (vitamin B5) is an essential molecule required for the synthesis of coenzyme A and acyl carrier protein (ACP). PanC (pantothenate synthetase) and PanD (aspartate-1-decarboxylase) involved in pantothenate biosynthesis.
6	VFG0431	52.91	8.00E-168	Vi antigen	*tviB*	Defensive virulence factors	Antiphagocytosis	Vi polysaccharide biosynthesis protein, UDP-glucose/GDP-mannose dehydrogenase. Prevent antibody-mediated opsonization, increase resistance

								to host peroxide and resistance to complement activation by the alternate pathway and complement-mediated lysis.
7	VFG0574	53.7	0	MgtBC	*mgtB*	Nonspecific virulence factor	Magnesium uptake system	Mg^{2+} transport protein. Hypothesized to be a magnesium transporter. MgtA and MgtB are not required for intracellular survival or for virulence. MgtC is essential for both functions.
8	VFG1395	40.09	6.00E-50	MgtC	*mgtC*	Nonspecific virulence factor	Magnesium uptake system	Homology to MgtC of *Salmonella enterica*, which is essential for the survival of *S. enterica* within macrophages. Magnesium acquisition.
9	VFG1864	45.45	5.00E-40	Mip	*mip*	Defensive virulence factors	Antiphagocyt osis	Macrophage infectivity potentiator (Mip). Necessary for intracellular survival. Belongs to the enzyme family of FK-506 binding proteins that exhibit the peptidyl-prolyl-cis/trans isomerase (PPIase) activity, catalyzes the isomerization of peptide bonds N-terminal to Pro residues.
10	VFG1971	44.01	1.00E-86	Capsule	*kpsF*	Defensive virulence factors	Phase variation	KpsF protein. Major antigenic component of the classic Penner serotyping system; Variation in the capsule structure may be caused by multiple mechanisms, such as exchange of capsular genes and entire clusters by horizontal transfer, gene duplication, deletion, fusion and the presence of

								homopolymeric G tracts in several *cps* genes. Play an important role in bacterial survival and persistence in the environment and evasion of host immune response; the presence of heptose residues in the capsule may be important for virulence. Heptose residues found in some cell surface-located glycoconjugates are required for adhesion.
11	VFG1214	40.53	5.00E-99	Type IV pili	*pilR*	Offensive virulence factors	Adherence	Two-component response regulator PilR. Involved in transcriptional regulation and chemosensory pathways that control the expression or activity of the twitching motility of the pili. Attaches to host cells, but not to mucin, causing a twitching motility that allows the bacteria to move along the cell surface; biofilm formation.
12	VFG1867	43.65	4.00E-58	SodB	*sodB*	Offensive virulence factors	Stress protein	Superoxide dismutase. A cytoplasmic iron superoxide dismutase; important for intracellular survival and transmission.
13	VFG1866	42.13	4.00E-63	RpoS	*rpoS*	Regulation of virulence-associated genes	Regulation	Stationary phase specific sigma factor RpoS. Stimulates intracellular replication and osmotic resistance and inhibits FliA-dependent traits.
14	VFG0670	40.21	9.00E-74	LPS	*gtrB*	Offensive	Host immune	Bactoprenol glucosyl transferase. Required for

						virulence factors	evasion	resistance to host defense and for the intracellular spread, but not for bacterial invasion.
15	VFG2361	45.65	1.00E-107	O-antigen	*galE*	Offensive virulence factors	Host immune evasion	UDP-glucose 4-epimerase. *Y. enterocolitica* O antigen expression is temperature regulated.<br>LPS O antigen mutants were severely impaired in their ability to colonize the Peyer's patches and did not colonize spleen and liver. The absence of O antigen in the outer membrane affects the expression of other Yersinia virulence factors.
16	VFG0036	45.13	3.00E-99	LPS	*bplC*	Offensive virulence factors	Endotoxin	Lipopolysaccharide biosynthesis protein. Required for the biosynthesis of trisaccharide to generate band A LPS. Prevents clearance of the organism by host surfactant protein; confers protection to the bacterium from complement-mediated cell lysis.
17	VFG1861	63.33	0	KatAB	*katA*	Offensive virulence factors	Stress protein	Catalase/(hydro)peroxidase. A periplasmic catalase, expressed maximally during the post-exponential phase; important for intracellular survival and transmission.
18	VFG0077	52.08	3.00E-72	ClpP	*clpP*	Offensive virulence factors	Stress protein	ATP-dependent Clp protease proteolytic subunit. Serine protease involved in proteolysis and is required for growth under stress conditions.
19	VFG1248	44.67	1.00E-65	Flagella	*fleQ*	Offensive	Adherence	Transcriptional regulator FleQ. Swimming motility;

						virulence factors		play a role in biofilm formation and other pathogenic adaptations.
20	VFG1381	60.38	0	Isocitrate lyase	*icl/aceA*	Defensive virulence factors	Cellular metabolism	Required for persistent infection. Isocitrate lyase is the initial enzyme in the glyoxylate shunt, a secondary metabolic pathway that allows bacteria to utilize fatty acids as carbon and energy sources when the availability of primary carbon sources is limiting; Isocitrate lyase, in combination with malate synthase, catalyzes the conversion of isocitrate to malate, a reaction that allows maintenance of the TCA cycle and synthesize carbohydrates from fatty acids.
21	VFG1855	65.91	0	Hsp60	*htpB*	Offensive virulence factors	Adherence	Hsp60, 60K heat shock protein HtpB. Mediate a complement-independent attachment to mammalian and amoebal host cells.
22	VFG1416	57.01	3.00E-40	PanC/Pan D	*panD*	Defensive virulence factors	Cellular metabolism	Lipid biosynthesis and metabolism play a pivotal role in the intracellular replication and persistence of *M. tuberculosis*. Pantothenic acid (vitamin B5) is an essential molecule required for the synthesis of coenzyme A and acyl carrier protein (ACP). PanC (pantothenate synthetase) and PanD (aspartate-1-decarboxylase) involved in pantothenate

								biosynthesis.
23	VFG1936	40	2.00E-45	LOS		Defensive virulence factors		Cj1135 - putative two-domain glycosyltransferase. LOS diversity is important for the ability to colonize a wide variety of hosts and intestinal niches.
24	VFG0037	40.79	9.00E-33	LPS	*bplB*	Offensive virulence factors	Endotoxin	Probable acetyltransferase. Required for the biosynthesis of trisaccharide to generate band A LPS. Prevents clearance of the organism by host surfactant protein; confers protection to the bacterium from complement-mediated cell lysis.
25	VFG0366	45.24	2.00E-66	Yersiniaba ctin	*ybtQ*	Nonspecific virulence factor	Iron uptake	Inner membrane ABC-transporter YbtQ. Iron-regulated genes. Ability to capture the iron molecules necessary for their systemic dissemination in the host.
